# Introduction and Application of the Interferon-γ Assay in the National Bovine Tuberculosis Control Program in South Korea

**DOI:** 10.3389/fvets.2020.00222

**Published:** 2020-04-28

**Authors:** Yun-Ho Jang, Tae-woon Kim, Min Kyu Jeong, Yoon Jeong Seo, Soyoon Ryoo, Chan Ho Park, Sin seok Kang, Young Ju Lee, Soon-Seek Yoon, Jae Myung Kim

**Affiliations:** ^1^Bacterial Disease Division, Animal and Plant Quarantine Agency, Gimcheon-si, South Korea; ^2^Foot and Mouth Disease Division, Animal and Plant Quarantine Agency, Gimcheon-si, South Korea; ^3^Gangwondo Livestock & Veterinary Service, Chuncheon-si, South Korea; ^4^Chungcheongbukdo Livestock & Veterinary Service, Chungju-si, South Korea; ^5^College of Veterinary Medicine, Kyungpook National University, Daegu, South Korea

**Keywords:** bovine tuberculosis, *Mycobacterium bovis*, IFN-γ, CFT, cattle

## Abstract

Bovine tuberculosis is a chronic disease impacting both public health and the livestock industry. The interferon (IFN)-γ assay has been introduced as an ancillary test for diagnosing bovine tuberculosis to overcome limitations of the skin test. The objective of this study was to assess the IFN-γ assay in terms of diagnostics and as a nationwide surveillance program in South Korea. From 2012 to 2013, cattle (*n* = 120) with bovine tuberculosis and cattle (*n* = 426) from bovine tuberculosis free herds were subjected to the IFN-γ assay to evaluate the sensitivity and specificity of the assay, respectively, depending on various cut-offs (0–3.5). When optical density of the cut-off was 0.1, the sensitivity and specificity were found to be 81.7% (74.7–88.6) and 99.5% (98.9–100.0), respectively. After introducing the IFN-γ assay as part of the national control program, the IFN-γ assay and single caudal fold skin test data were collected from 47 regional veterinary services to compare the results of these two tests. Overall, the agreement between the IFN-γ assay and the single caudal fold skin test (*n* = 492,068) was 98.2%, and Cohen's kappa value for the two methods was 0.47. Serial and parallel use of the IFN-γ assay and skin test for the bovine tuberculosis control program were compared using samples (*n* = 91) from cattle confirmed as bovine tuberculosis positive in laboratories from 2014 to 2016. Parallel screening for bTB showed much higher sensitivity (86/91, 94.5%) than the following screening approaches: serial (47.2%, 43/91), single screening using CFT (63.7%, 58/91), or the IFN-γ assay (78.0%, 71/91). These results indicate that the IFN-γ assay and single caudal fold skin test are complementary to each other; therefore, parallel use of these two tests is considered a useful approach to reduce the prevalence of bovine tuberculosis in South Korea.

## Introduction

Bovine tuberculosis (bTB), caused primarily by *Mycobacterium bovis (M. bovis)*, is a chronic disease impacting both public health and the livestock industry. Worldwide, eradication programs for bTB are based on detection of infected animals and their removal from the herd (test and slaughter) (1–3). In South Korea, although this strategy has been used for over half a century, bTB continues to be endemic. Diagnostic tests for bTB are mainly based on the cell-mediated immune (CMI) response, and the tuberculin skin test is a widely used standard screening method worldwide (2). However, the tuberculin skin test has some limitations, such as subjectivity in interpretation of results, requirement of a skilled technician, and false-positive reactions caused by sensitization of other mycobacteria or by local inflammation (4). The interferon gamma (IFN-γ) assay, used as an ancillary test for bTB diagnosis, has improved the sensitivity of bTB testing (5). This assay is advantageous over the tuberculin skin test because it enables early detection of *M. bovis* infections in animals without revisits, and this prevents variation in observation when assessing skin reactions (6). The IFN-γ assay has been approved for use in a number of national programs for bTB control in many other countries including European Union, USA, New Zealand, and Australia (7, 8).

The number of dairy and beef cattle in the South Korea have been maintained around 400,000 and 2,700,700 for several years (9). A different surveillance program for bTB has been used for beef vs. dairy cattle in South Korea (10). All dairy cattle over 12 months old should be tested annually by means of the caudal fold test (CFT) using purified protein derivative of *M. bovis* (PPD-B) (CAVAC, South Korea). In contrast, there was no regular testing for beef cattle except for slaughter house surveillance. A pilot monitoring project using the IFN-γ assay for beef cattle was undertaken from 2014 to November 2016. Approximately 300,000 beef cattle a year, raised at farms with a history of bTB outbreaks, were tested using a commercial IFN-γ kit, TB feron ELISA (Bionote, South Korea). Since November 2016, pre-movement test prior to farm to farm trade has been adopted as a mandatory policy for beef cattle; however, this is not the case for dairy cattle due to their regular testing. Since then, the IFN-γ assay has been adopted as the primary test along with the skin test to diagnose bTB. Both types of cattle farms with ongoing outbreaks should be tested every 2 months by parallel testing (CFT and IFN-γ assay) concurrently until all cattle are identified as not reactive in two successive tests. During this period, cattle movement from outbreak herds is strictly restricted except for movement to slaughter houses. The two primary tests for bTB diagnosis should only be carried out by state veterinarians in South Korea. The objective of this study was to assess the efficacy of the IFN-γ assay in terms of diagnostics and as a large-scale surveillance program in South Korea.

## Materials and Methods

### Use of the IFN-γ Assay

#### Samples

Heparinized blood and tissue samples, including lung, and cervicothoracic lymph nodes, were collected from 2012 to 2013. All cattle were examined for visible lesions by veterinarians, and tissue samples sent to the laboratory for bacteriological and pathological examination to identify infection status. Samples for determining the specificity (Sp) of the assay were collected at a slaughter house located in an OIE certified bTB free region in South Korea. In total, 426 cattle were identified as being bTB negative by laboratory tests, and results from the IFN-γ assay of these cattle were used to determine the cut-off as a true negative. Samples for determining sensitivity (Se) of the assay were collected from cattle diagnosed as positive by the CFT from herds in regions with bTB outbreaks. In total, 120 cattle identified as *M. bovis* infected from tissues and/or identified by polymerase chain reaction (PCR) after detection of typical bTB lesions on gross/microscopic examination were considered as truly bTB-positive (11, 12).

#### IFN-γ Assay

Whole blood samples were collected in sodium-heparinized tubes from the jugular vein of cattle and transferred to the laboratory at room temperature (18–25°C) on the day of collection. All blood samples were stimulated within 10 h after collection. In the laboratory, blood samples were divided into three aliquots of 1.5 ml each in 24 well plates. Commercial PPD-B, PPD-A (purified protein derivative of *M. avium)* (CZ veterinaria, Spain), and PBS (phosphate buffered saline) as a nil antigen control were used for stimulation of immune response in whole blood samples. One hundred microliters of PPD-B (0.3 μg/ml), PPD-A (0.3 μg/ml), or PBS were added to and mixed in each well and incubated at 37°C in a CO_2_ incubator for 24 h. After incubation, 24 well plates were centrifuged for 20 min at 500 × g, and plasma from the upper layer was harvested. Plasma samples were tested in duplicate with a sandwich enzyme immunoassay using the TB feron ELISA (Bionote, South Korea) per the manufacturer's instructions. Optical density (O.D) of each well was measured at 450 nm. The mean O.D of each sample was calculated and used to define the cut-off values. The O.D of a sample stimulated with PPD-B minus the O.D of a sample stimulated with PPD-A (O.D_PPD−B_-O.D_PPD−A_), and O.D of a sample stimulated with PPD-B minus the O.D of nil antigen treated sample (O.D_PPD−B_-O.D_PBS_) values were used as cut-off criteria.

### Statistical Analysis

The Receiver Operating Characteristic (ROC) curve, indicating the Se and Sp of the assay depending on each cut-off value, was determined using STATA 15 (StataCorp, USA). Se and Sp were plotted for various cut-off values (0–3.5) to construct the ROC curve. Area under the curve (AUC) for the ROC curve was calculated to define the diagnostic utility of the IFN-γ assay examined in this study. AUC analysis indicated that 0.5 < AUC ≤ 0.7 represents low accuracy, 0.7 < AUC ≤ 0.9 moderate accuracy, and 0.9 < AUC ≤ 1.0 represents high accuracy (13).

### Bacteriological Culture and Identification Using Polymerase Chain Reaction

Five to ten grams of all tissue samples collected at postmortem examination were transferred into sterile stainless-steel containers, cut into small pieces using sterile scissors, and then liquefied in 10 ml of PBS using a homogenizer (Tokken, Chiba, Japan). Homogenized tissues were transferred to centrifuge tubes and an equal volume of 10% oxalic acid was added. Samples were then incubated at room temperature for 10 min to decontaminate tissue samples. Tissue solutions were filtered using 70 & 40 μm cell strainers step by step (Thermo Fisher Scientific, MA, USA) and filtered samples were centrifuged at 3,000 × g for 10 min. The supernatant was decanted and the pellet was re-suspended in 2 ml PBS and vortexed strongly. Finally, tissue suspensions were inoculated into Mycobacteria growth indicator tubes (MGIT) (Becton, Dickinson (BD) and company, NJ, USA), 7H11 slant agar (BD), and Lowenstein Jensen slant agar (BD), and incubated at 37°C for 4 weeks.

DNA was extracted from suspected bTB colonies for identification by polymerase chain reaction (PCR) using the DNeasy Blood and Tissue kit (Qiagen, Germany) according to the manufacturer's instructions. Primers were designed targeting the insertion elements (IS), IS1081 and IS6110, which specifically exist within the genome of *Mycobacterium bovis* (7, 14, 15). Multiplex PCR was performed for IS1081 and IS6110 in a reaction mixture (20 μl) (Table 1) containing 10 pmol of each primer, 14 μl AccuPower hotstart PCR premix (Bioneer, South Korea), and 2 μl DNA template. PCR conditions were as follows: 94°C for 5 min, followed by 35 cycles of 94°C for 30 s, 65°C for 30 s min, 72°C for 1 min, with a final extension at 72°C for 5 min. PCR products were analyzed using agarose gel electrophoresis with a 1.5% agarose gel. When one or two PCR bands corresponding to the two sets of primers were observed, they were considered to be *M. bovis* DNA (see Figure S1).

**Table 1:** Primers used in this study.

**Target gene**		**Primer Sequence (5^**′**^->3^**′**^)**	**Product size**
IS6110	Forward	CCAGATGCACCGTCGAACGGCTGAT	397 bp
	Reverse	CGCTCGCTGAACCGGATCGATGTGT	
IS1081	Forward	GGCAGCTATTTCCCGGACTGGCTG	135 bp
	Reverse	CACACCAAGTGTTTCGACCAGGCGC	

### Introduction and Application of the IFN-γ Assay as Part of the bTB Control Program in South Korea

#### Agreement Between the IFN-γ Assay and CFT

From 2014 to 2017, a total of 1,678,291 and 1,716,179 cattle were tested by 47 regional veterinary services in South Korea using the IFN-γ assay and CFT, respectively. Of these, 8,157 and 8,949 cattle were diagnosed as positive by the two tests, respectively. Of the total tested cattle, results from 492,068 cattle that were tested concurrently using these two methods were analyzed for agreement using Cohen's Kappa value (16).

### Evaluation of Parallel Use of the IFN-γ Assay and the CFT in Herds With Ongoing bTB Outbreaks

From 2014 to 2016, 116 cattle from herds with bTB tested concurrently using both the CFT and IFN-γ assay were used to verify that parallel use of the two tests can effectively eliminate reactors from herds. Results from these two screening tests, gross/microscopic examination, acid fast staining, bacteriological culture, and direct PCR from tissue samples were compared. The cut-off for the IFN-γ assay was >O.D 0.1 (450 nm) for O.D_PPD−B_-O.D_PPD−A_ and O.D_PPD−B_-O.D_PBS_. Gross/microscopic examinations were conducted by a veterinary pathologist to detect typical lesions such as tubercle, granuloma with central caseation necrosis, and acid-fast staining. Additionally, culture & tissue direct PCR was performed according to the method mentioned in section Bacteriological culture and identification using polymerase chain reaction. Residual homogenized tissues, after bacterial culture, were pretreated as follows for direct PCR: incubation at 37°C for 30 min after the addition of 180 μl of lysozyme (20 μl/ml, Sigma-Aldrich, USA), followed by incubation at 56°C for 60 min after the addition of 25 μl of proteinase K (Sigma-Aldrich, USA). DNA was extracted from the lysed tissue samples using the NucleoSpin® Soil kit (Macherey Nagel, Germany) according to the manufacturer's instructions.

## Results

### Optimal Test Cut-Off for the IFN-γ Assay

Se (*n* = 120) and Sp (*n* = 426) of the IFN-γ assay were calculated based on cut-off values ranging from 0 to 3.5. When cut-off O.D for the IFN-γ assay was 0.1, Se and Sp were 81.7% (95% CI, 74.7–88.6) and 99.5% (95% CI, 98.9–100.0), respectively (Table 2). When cut-off O.D was 0.05, the assay showed higher sensitivity (86.7, 95% CI, 80.6–92.8); however, Sp was found to be lower (98.4, 95% CI, 97.2–99.6) than when O.D was 0.1. The AUC of this ROC curve was 0.9891.

**Table 2:** Sensitivity and specificity of the IFN-γ assay depending on the cut-off value.

**Criteria**		**Cut-off**	**Se%**	**Sp%**
OD_PPD−B_-OD_PPDA_ and OD_PPDB_-OD_PBS_		0	91.7	82.9
		0.005	90.0	91.5
		0.01	89.2	94.1
		0.0125	89.2	94.4
		0.025	86.7	96.9
		**0.05**	**86.7**	**98.4**
		**0.1**	**81.7**	**99.5**
		0.2	74.2	100.0
		0.3	69.2	100.0
		0.4	65.0	100.0
		0.5	60.0	100.0
		0.6	51.7	100.0
		0.7	45.0	100.0
		0.8	44.2	100.0
		0.9	42.5	100.0
		1	37.5	100.0
		1.5	20.0	100.0
		2	10.0	100.0
		2.5	4.2	100.0
		3	0.8	100.0
		3.5	0.0	100.0
	**Cut-off** **=** **0.05**			
	**Positive**	**Negative**	**Total**	**Percentage (95% CI)**
bTB infected	104	16	120	Se: 86.7% (80.6–92.8)
Control	7	419	426	Sp: 98.4% (97.2–99.6)
	**Cut-off** **=** **0.1**			
	**Positive**	**Negative**	**Total**	**Percentage (95% CI)**
bTB infected	98	22	120	Se: 81.7% (74.7–88.6)
Control	2	424	426	Sp: 99.5% (98.9–100.0)
AUC	0.9891			0.9814–0.9969

### Agreement Between the IFN-γ Assay and CFT, and Evaluation of Parallel Use of the Two Tests

**Figure 1:**
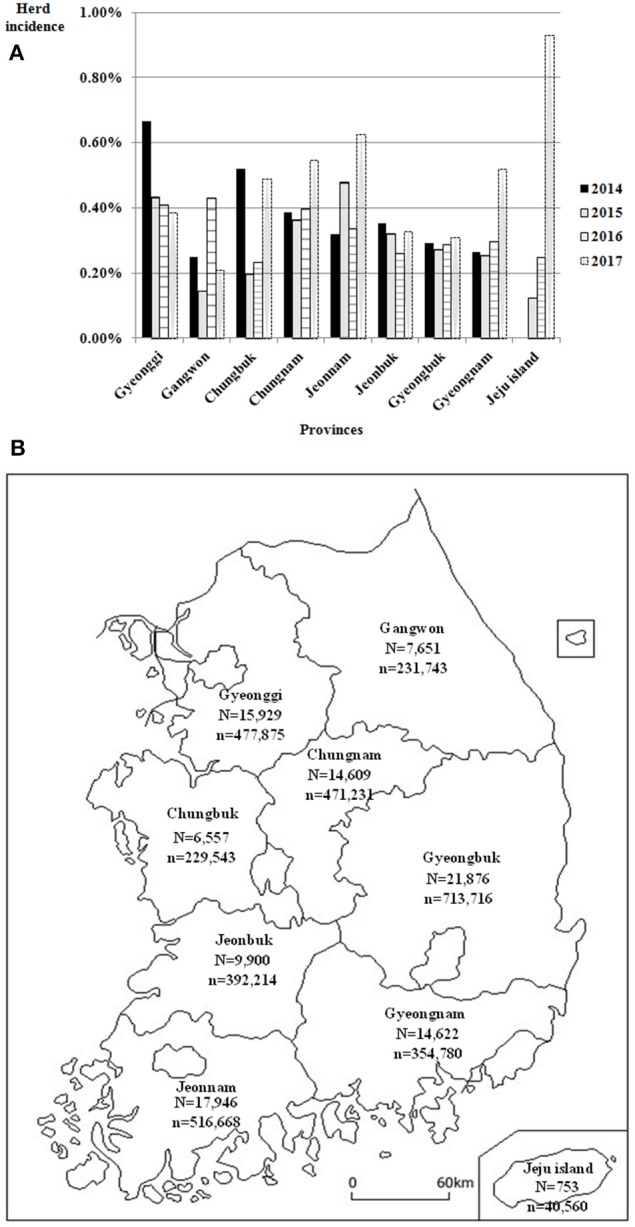
**(A)** Regional herd incidence in nine provinces of South Korea from 2014 to 2017. **(B)** Location of these nine provinces of South Korea and number of cattle herds and heads in each province in 2017. *N*, No. of cattle herds; *n*, No. of cattle heads.

Agreement between results of the IFN-γ assay and CFT for 492,068 cattle tested from 2014 to 2017 is shown in Table 3. The overall agreement of the two tests was markedly high (98.2%); however, Cohen's kappa value for the two methods was 0.47, indicating moderate agreement. Since the introduction of the IFN-γ test as one of the elements of the control program, the rate of detection of bTB from 2014 to 2017 in South Korea has decreased continuously from 1.45 to 0.42%, but the herd incidence has remained at around 0.3% (Table 4). The regional detection rate for most regions has decreased from 2014 to 2017, similar to nationwide figures; however, regional herd incidence was found to fluctuate during the same period except on Jeju Island and in Gyeongnam province where the number of bTB outbreaks increased profoundly in 2017 (Figure 1).

**Table 3:** Agreement between the skin test and IFN-γ assay.

		**Skin test (CFT)**	
		**Negative**	**Positive**
IFN-γ	Negative	479,412	3,849
	Positive	4,772	4,035
Overall agreement	98.2% (98.2–98.3)		
Kappa value	0.47		

*Suspicious results from CFT were excluded in this calculation due to very low percentage (0.09%).

**Table 4:** Change in herd incidence and detection rate from 2014 to 2017.

	**2014**	**2015**	**2016**	**2017**
No. of tested herds	25,866	26,368	43,012	99,044
No. of infected herds	376	284	337	414
No. of total herds	123,288	113,484	109,016	105,073
Herd detection rate^a^	1.45%	1.08%	0.78%	0.42%
Herd incidence	0.30%	0.25%	0.31%	0.39%

a *Herd detection rate: No. of infected herds/No. of tested herds*.

After the introduction of the IFN-γ assay as part of the national control program from 2014 to 2016, 91 out of a total of 116 samples collected from herds with ongoing bTB outbreak were confirmed as bTB positive by *M. bovis* culture and/or typical bTB lesions. Positive samples were most frequently found using the IFN-γ assay (71/91, 78.0%), followed by IS1081 PCR (76.9%), gross/microscopic examination (70.3%), CFT (63.7%), *M. bovis* isolation (58.2%), IS6110 PCR (51.6%), and acid-fast staining (29.7%). Differences in detection efficiency for bTB depending on the application of the two tests are shown in Table 5. Parallel screening for bTB showed much higher Se (86/91, 94.5%) than the following screening approaches: serial (47.2%, 43/91), single screening using CFT (63.7%, 58/91), or the IFN-γ assay (78.0%, 71/91). When true infection was strictly defined using *M. bovis* cultures (*n* = 53), normally used as a gold standard test to evaluate diagnostic tools, gross/ microscopic examination showed the highest Se (46/53, 86.8%), followed by the IFN-γ assay (44/53, 83.0%), IS1081 PCR (36/53, 67.9%), IS6110 PCR (30/53, 56.6%), CFT (29/53, 54.7%), and acid-fast staining (24/53, 45.3%).

**Table 5:** Difference in sensitivity depending on application of the control program.

**Control program**		**No. of positive by laboratory tests**
**CFT**	**IFN-γ**	
+	+	43
+	–	15
–	+	26
–	–	5
±	+	2
±	–	0
Total		91
**Application of control program**		**Se**
Parallel screening (CFT or IFN-γ)		94.5% (86/91)
Single screening (by IFN-γ)		78.0% (71/91)
Single screening (by CFT)		63.7% (58/91)
Serial screening (by CFT+IFN-γ)		47.2% (43/91)

## Discussion

The IFN-γ assay is designed to measure the CMI, the dominant immunological response to tuberculosis in cattle and is mainly used as an ancillary test (2). In this study, we attempted to assess the efficacy of the IFN-γ assay as a diagnostic tool and large-scale surveillance program for detection of bTB in South Korea.

Generally, the calculation formula for the IFN-γ assay is based on the difference between the titers of IFN-γ that exist in each sensitized plasma sample using three kinds of stimulating materials, including bovine PPD, avian PPD, and PBS (O.D_PPD−B_-O.D_PPD−A_, O.D_PPD−B_-O.D_PBS_, O.D_PPD−B_ > O.D_PPD−A_ or O.D_PPD−B_-O.D_PPD−A_ / O.D_positivecontrol_-O.D_negativecontrol_). This approach has been used depending on the epidemiological context in various countries or regions (6, 8, 17). This calculation using PPDs accounts for sensitization by non-tuberculous organisms such as *M. avium* and IFN-γ in blood released for other reasons (6). Additionally, defined mycobacterial antigens, including ESAT-6 and CFP-10, are also being used to increase Sp of results in regions with low bTB prevalence or officially bTB free (OTF) countries (17, 18). The selected cut-off value is one important factor in the application of the IFN-γ test. The cut-off value for this study is estimated at O.D 0.1 for Se 81.7% (74.7–88.6) and Sp of 99.5% (98.9–100.0), whereas the cut-off value at O.D 0.05 provides Se of 86.7% (80.6–92.8) and Sp of 98.5% (97.2–99.6). As the cut-off value changes, Se and Sp of the IFN-γ assay will vary in opposing directions; thus, the analysis criteria may require adaptation depending on the region's infection status and the test application conditions (8, 19). Previous studies have reported the range of Se for the IFN-γ assay to be between 73.0 and 100.0% and showed that Sp ranged from 85.0 to 99.6% (6, 17, 20). These results were likely influenced by several factors, such as cattle population, cut-off values, the source of PPD tuberculin, and the animals defined as the “gold standard” (6). The AUC of ROC indicated that this IFN-γ assay has good power of discrimination to be used for diagnosis of bTB. Although bTB has been endemic in South Korea since first reported in 1913, recently, herd incidence for bTB in South Korea has not been very high (about 0.3%) (10). In terms of Sp, a 1% difference implies that there would be over 10,000 false positive cattle from one million cattle tests per annum. Consequently ~100 million dollars would be required for compensation and slaughter of uninfected cattle diagnosed as false positives. Therefore, 0.1 as a cut-off value obtained using the formula devised using PPDs is regarded as adequate to control bTB in South Korea. Since the introduction of the bTB eradication program, bTB testing by private veterinarians has not been allowed in South Korea. Moreover, the number of state veterinarians has been insufficient to perform the CFT as a pre-movement test or as a regular interval test for all cattle. Consequently, the CFT has only been used for parallel testing in outbreak herds and in regular interval testing for dairy cattle, which have a population one-seventh the size of beef cattle. The IFN-γ assay was introduced to reinforce the control policy of bTB as a screening test for beef cattle, which were monitored only by slaughterhouse surveillance, in spite of their larger population size relative to dairy cattle. However, many studies on the IFN-γ assay have reported that it is appropriate as an ancillary test in herds exposed to bTB due to its higher Se and lower Sp than the skin test (6, 8). Hence, long term monitoring of the IFN-γ assay as a primary test should be conducted, although Sp was very high in South Korea from our results.

Our results showed high agreement (98.2%) on identification of most cattle as bTB negative using both the skin test and IFN-γ assay; however, the results had a relatively low Cohen's kappa value (0.47) caused by diagnosis of some cattle as bTB positive based on only one kind of test (CFT-positive and IFN-γ assay-negative or CFT-negative and IFN-γ assay-positive (21, 22). Several other studies also reported agreement between the two tests at various levels. In a study conducted in Brazil, kappa values were 0.54–0.66 depending on time points when the IFN-γ assay (Bovigam, criteria for the IFN-γ assay not provided) was performed. Agreement increased to 0.649–0.713 when data from cattle affected by *M. avium* complex were withdrawn (23). A study performed in Ireland in 2015 showed relatively higher agreement between the single intradermal comparative tuberculin test and IFN-γ assay (Bovigam, cut-off for the INF-γ assay = 0.05) (20). Contrastingly, a study from dairy cattle in southern Chile showed a kappa value of ~0.3, indicating fair agreement in efficacy between the two tests when the cut off for the IFN-γ assay (Bovigam) was 0.05 or 0.1 (24). Most instances of low agreement resulted from false negatives obtained due to the diagnostic methods used. Potential causes of identification of false negatives using the skin test are as follows: difficulty of intra-cutaneous injection of the PPD (too superficial or too deep), taking observations too early after injection (reading the result of the skin test 72 h after injection is recommended), error in recording the thickness of skin, problems arising from the tuberculin used (expired or inadequately stored or manufacturing errors), desensitization, and reduction of immune response due to co-infection with *Fasciola hepatica* (6, 25, 26). Two different PPD-B preparations, manufactured by different companies, were used for skin test (CAVAC) and IFN-γ assay (CZ veterinaria) in South Korea. These two companies don't provide the potency of PPD-B as international unit (IU) but as protein concentration (CAVAC-0.2 mg/0.1 ml and CZ veterinaria-0.3 μg/ml). Therefore, the potency of the two PPD-B preparations may be not equivalent, although each stimulating agent is standardized through comparison with reference material. This difference of potency of PPD-B may also be one of the causes of the low agreement between the two tests. But analysis for potency of two commercial PPD-B preparations is not included in this study. Other than the false negative results mentioned above, it is probable that the IFN-γ assay could detect the immune response for bTB earlier than the skin test (6, 20, 21). In contrast, one of the potential causes of false negative diagnosis using the IFN-γ assay might be incorrect blood status, showing an overall reduction in titer by degradation of T lymphocyte activity, which is associated with the time required for transport or temperature of storage. Several studies reported that delays in the processing of blood and improper temperatures, which might be encountered in field situations or upon delivery, might impact IFN-γ production (27, 28). Some field veterinarians in South Korea prefer the caudal vein over the jugular vein to collect blood due to the relatively easier restraint of cattle for caudal vein blood collections. However, the smaller diameter of the caudal vein vs. that of the jugular vein might cause blood cell damage and micro-clotting, which could lead to the capture of lymphocytes, lowering IFN-γ production (29). Co-infection or pre-sensitization with other organisms, such as *M. avium* subsp. *paratuberculosis* could also impact the result showing O.D_PPD−B_-O.D_PBS_ higher than the cut-off value; however, O.D_PPD−B_-O.D_PPD−A_ lower than the cut-off value. Some studies reported that Se of the IFN-γ assay was reduced in the group of cattle co-infected with bTB and Johne's disease (30, 31). In South Korea, there is no regular surveillance program for paratuberculosis. However, some studies reported that the sero-prevalence of paratuberculosis in South Korean cattle ranged from 3.3 to 7.1% (32). Therefore, it is possible that the diagnosis of bTB by the IFN-γ assay is likely to be influenced by co-infection or pre-sensitization, although additional investigations are needed to confirm this. Similar to other studies, our data, obtained from large-scale surveillance, also support the results of the skin test, and found that the IFN-γ assay could detect slightly different sub-populations of bTB infected cattle due to the abovementioned reasons, even though both tests use the host's CMI response (6, 33).

After the introduction of the IFN-γ assay in South Korea, the herd detection rate for bTB continuously decreased because the number of tested herds markedly increased as compared to the period before this assay was introduced. However, herd incidence increased slightly from 2015 to 2017. This was speculated to be due to the fact that the IFN-γ assay could detect more reactor cattle recently in the regions of South Korea where there was no field surveillance for beef cattle, except slaughterhouse surveillance until 2013. Regional differences in herd incidence were not significant from 2014 to 2017 because cattle trades between regions are common in South Korea. Moreover, there was no obvious correlation between regional incidence and size of cattle herds or heads. On account of frequent inter-regional movement and unclear regional differences in herd incidence for bTB, differential regional application of detailed parts of the control program, such as testing interval and frequency, which are operational in other countries, have not yet been considered in South Korea (34–36).

Many studies have stated that parallel testing using a combination of two different tests could improve Se of assays and accelerate the resolution of a TB outbreak (6, 8, 12, 37). In this study, when the two kinds of tests were used as single, serial, or in parallel for reactors identified using several laboratory tests, the parallel screening showed the highest Se (86/91, 94.5%). Thus, parallel screening using the IFN-γ assay and skin test based on CMI could be a suitable strategy to eliminate reactors from bTB herds, as already reported in previous studies.

In South Korea, although the control program based on the CFT and meat inspection has been conducted over the last 50 years since the first bTB detection in 1913, bTB is still regarded as an endemic disease, which greatly affects cattle husbandry. The IFN-γ assay was introduced to overcome some disadvantages of the skin test and is currently used as an ancillary test to complement the skin test in the EU, USA, New Zealand, and Australia (4, 20, 33). Unlike most countries that adapted the IFN-γ assay as a supplementary test, in South Korea, it is included in the bTB surveillance program as a primary test equivalent to the skin test. This is the first study to describe the nationwide surveillance of bTB using the IFN-γ assay in South Korea. Our study revealed a change of bTB incidence after the introduction of the IFN-γ assay and its agreement with the skin test as a diagnostic method. Furthermore, our study showed that parallel use of the IFN-γ assay and skin test for bTB outbreak herds may serve as an efficient strategy in field conditions in South Korea.

## Data Availability Statement

All datasets generated for this study are included in the article/Supplementary Material.

## Ethics Statement

We confirmed that there is no requirement to obtain the approval for research on animals slaughtered due to infectious diseases from IACUC (Institutional Animal Care and Use Committee) in South Korea.

## Author Contributions

Y-HJ performed the statistical analyses. Y-HJ and JK drafted the manuscript. Y-HJ, YL, S-SY, and JK designed the study and coordinated the work. CP and SK collected clinical samples and conducted gross and microscopic examinations. Y-HJ, SR, TK, MJ, and YS participated in the laboratory experiments, and generation and collection of the data, and collaborated in the interpretation of the results. All authors critically revised the manuscript. All authors read and approved the final manuscript.

## Conflict of Interest

Y-HJ was employed by the Animal and Plant Quarantine Agency (government-affiliated), and CP and SK were employed by the Regional Government Institute (veterinary service). The remaining authors, including CP and SK, declare that the research was conducted in the absence of any commercial or financial relationships that could be construed as a potential conflict of interest.

## Supplementary Material

Figure S1 PCR result for identification of *M. bovis*. Line M: DNA molecular weight marker (100 bp); Line 1~2: *M. bovis* isolates; Line 3: *M. intracellulare* ATCC 13950, Line 4: *M. avium* subsp. *hominissuis* 104, Line 5: *M. avium* subsp*. paratuberculosis* ATCC 19698; Line -: negative control, Line +: *M. bovis* AN5.
