# Supplementary figures and images for: Introduction and Application of the Interferon-γ Assay in the National Bovine Tuberculosis Control Program in South Korea
Source: Front Vet Sci. 2020 Apr 28;7:222. doi: 10.3389/fvets.2020.00222 (PMC7198753; doi:10.3389/fvets.2020.00222)

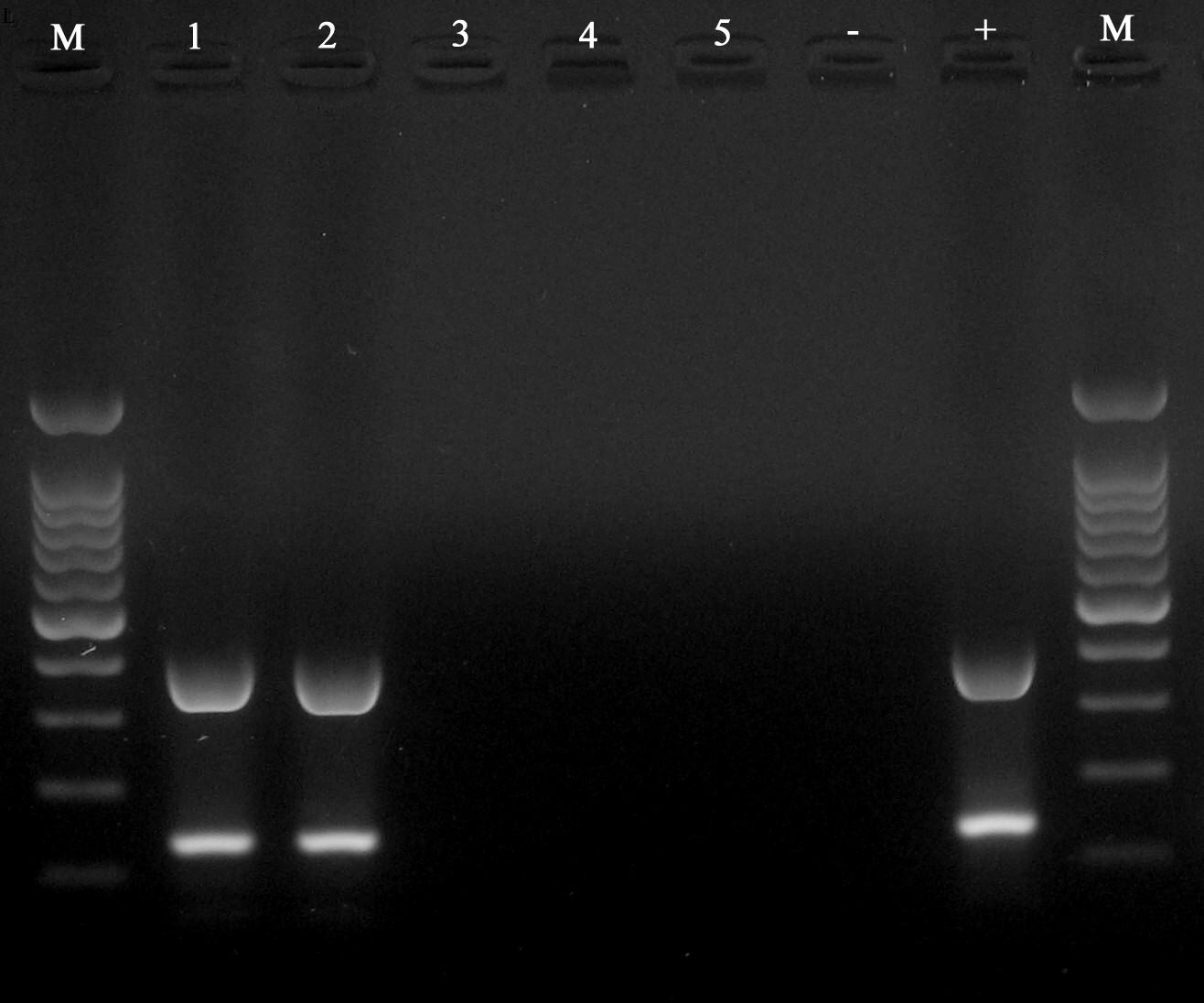

Supplement: Figure S1 — PCR result for identification of M. bovis. Line M: DNA molecular weight marker (100 bp); Line 1~2: M. bovis isolates; Line 3: M. intracellulare ATCC 13950, Line 4: M. avium subsp. hominissuis 104, Line 5: M. avium subsp. paratuberculosis ATCC 19698; Line -: negative control, Line +: M. bovis AN5. [file Image_1.TIF]
